# Direct Femtosecond Laser Writing of Micro-Optical Components

**DOI:** 10.3390/mi16101142

**Published:** 2025-10-04

**Authors:** Alessandra Nardini, Rebeca Martínez Vázquez, Behjat Sadat Kariman

**Affiliations:** 1 Department of Experimental Medicine, University of Salento c/o College Isufi, Centro Ecoteckne, Via Monteroni, 73100 Lecce, Italy; alessandranardini@cnr.it; 2 Institute for Photonics and Nanotechnologies (IFN), National Research Council, Piazza L. da Vinci 32, 20133 Milan, Italy; 3 Department of Physics, Politecnico di Milano, Buiding 8, Piazza L. da Vinci 32, 20133 Milan, Italy; behjatsadat.kariman@polimi.it

Direct femtosecond laser writing (DLW), also known as two-photon polymerization (2PP), emerged as a true 3D micro/nano-structuring method in 1997 when Mauro and co-workers first demonstrated infrared femtosecond laser photopolymerization of a UV-curable resist. They exploited the quadratic intensity dependence of two-photon absorption to confine polymerization into a voxel with sub-diffraction dimensions [1]. The underlying process, i.e., non-linear absorption at the focus, threshold radical (or cationic) chain growth, and diffusion-limited termination, favors the high spatial selectivity and aspect ratio control of the technique. Within a few years of that first publication, the same group further developed the technique by demonstrating functional micro-oscillators with features down to the 120 nm size, thus establishing the unmatched resolution and design freedom of DLW [2].

While fast improvements have been favored by the scaling laws that link the voxel size with physical parameters like the focusing optics numerical aperture, laser repetition rate and translation speed, pushing resolution below 100 nm [3], practical performance is set as much by the photochemistry as by optics [4]. Regarding chemistry, DLW leverages classic free-radical acrylate systems and epoxide-based (cationic) resists, as well as hybrid organic–inorganic sol–gel matrices; it also features photoresist formulation, the oxygen concentration, and post-cure strategies tune shrinkage, mechanics, and surface functionality of the obtained structures [5]. Presently, there is a broad range of available photoresists—from hard polyacrylics and epoxides (including SU-8 variants) to hydrogels and custom monomer mixtures—alongside dedicated two-photon photo-initiators and sensitization strategies, linking chemistry to achievable voxel geometry, throughput, and biocompatibility [4,5,6]. Overall, these laser and chemistry developments trace a clear line from the original DLW proof-of-concept to today’s fine-feature, materials-diverse direct-laser-writing platforms.

**Figure 1:**
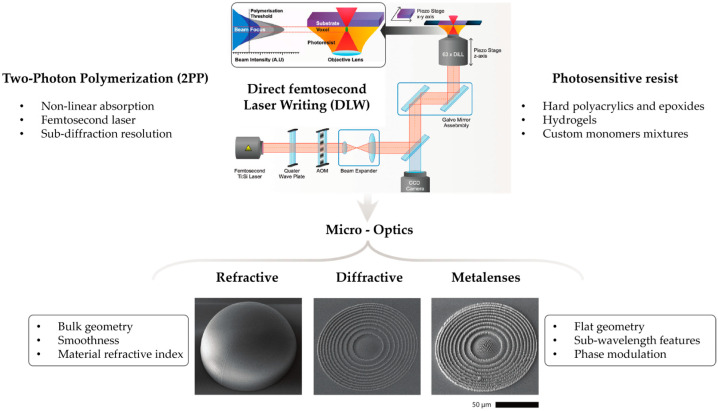
Direct femtosecond laser writing for micro-optics fabrication. Adapted from [5,18].

Lenses are fundamental optical components that can be used individually or in arrays to manipulate light. Through careful design, they enable the modulation of incident light in various ways, including diffusion, beam shaping, intensity equalization, and optical focusing [7]. These capabilities facilitate advanced functionalities that are often challenging to achieve with traditional optics. Lenses are typically classified based on their aperture geometry into three main types, namely rectangular, hexagonal, and circular, each offering distinct advantages in performance and fabrication [7,8]. Among these, circular refractive and diffractive lenses are the most widely used due to their optical symmetry, thus supplying more efficient manufacturing processes. When scaled down to the microscale, optimal performance in refractive microlenses depends on a continuous and uniform surface profile, which directly influences light propagation, minimizes scattering losses, and ensures high-quality optical focusing [9]. DLW has significantly advanced the production of refractive and diffractive micro-optic lenses [7,8,9]. This technique enables submicron-scale three-dimensional structuring, offering precise control over geometry, optical functionality, and refractive index distribution. DLW can be performed in immersion, dip-in, or hybrid modes. In immersion mode, a medium is introduced between the objective lens and the substrate, enabling exposure of the photoresist on the substrate’s back side [8,9,10,11]. In contrast, dip-in mode facilitates direct structuring on the front side by placing the photoresist in direct contact with the objective lens [8,9,10,11]. Figure 1 illustrates an overview of DLW principles together with the main micro-optics examples that will be discussed within this editorial.

Recent advancements have demonstrated the versatility of DLW in complex lens architectures. Schmid et al. utilized a combined approach with the photoresist IP-Dip to fabricate aligned aspheric lenses on both sides of a transparent substrate in a single writing step. Their method offers several advantages, including alignment-free integration of different photoresists, high structural precision, and flexible compact design [11]. The successful fabrication of doublet objectives printed on opposing surfaces demonstrated DLW’s capability for miniaturized, multifunctional optics. These innovations underscore its growing relevance in biomedical imaging, photonic integration, and lab-on-a-chip technologies.

Beyond structural precision, laser exposure conditions in DLW significantly influence not only the dimensions of fabricated features but also their optical properties, particularly the refractive index, which plays a crucial role in optical performance [8,12]. The single-step fabrication of 3D micro-optics with tunable refractive indices was demonstrated by varying the femtosecond exposure dose. Using hybrid photoresists (SZ2080), they fabricated spherical microlenses while precisely modulating material density and morphology, enabling direct manipulation of the refractive index during fabrication. The exposure intensities ranged from 0.237 to 0.284 TW/cm^2^ [12]. This approach provides enhanced design flexibility for multifunctional, free-form micro-optical elements operating across broad spectral ranges. Material composition also plays a role in expanding optical functionality. Most commercial optical photoresists used in femtosecond laser-based fabrication are transparent, which limits optical design flexibility [13]. In 2023, a method was introduced for fabricating colored micro-elements using DLW, broadening the design possibilities of 3D-printed optical components by incorporating pigments or dyes into commercially available photoresists or by dyeing structures post-fabrication [13]. This enables integrated spectral filtering and expands applications in hyperspectral imaging and multifunctional photonic devices. These findings emphasize the importance of surface quality in optical imaging. Ultra-smooth surfaces are vital for optical imaging through refractive microlenses, where the gold standard for surface smoothness is less than λ/2 to minimize scattering and ensure high-resolution performance [9,14]. Reaching this level of precision demands careful optimization of both design and fabrication processes. According to the methodology described in [14], quasi-parabolic microlenses with tunable focal lengths were produced using a hybrid DLW approach combining grayscale two-photon polymerization and a three-step UV crosslinking process. Gradual laser power reduction minimizes voxel edge effects, resulting in smooth surfaces, while using a biocompatible photoresist (SZ2080) enables potential biological applications for instance, in vivo imaging [14]. Taken together, micro-optic refractive lenses fabricated through this high-resolution laser-based technique, are increasingly applied in photonic systems such as fiber-optic communications, bio-imaging, networks, and biomedical devices [7,14,15], where their precise tunability and structural versatility are essential.

Unlike refractive microlenses, which rely on continuous thickness modulation and thus require bulky geometries, diffractive and metasurface-based lenses achieve wavefront control through engineered subwavelength features. This approach not only reduces device footprint but also allows the precise manipulation of the phase, amplitude, and polarization of the incident light at the microscale. Compared to their refractive counterparts, such flat optics minimize alignment issues, support higher level of integration, and open the way to multifunctional, lightweight, and compact photonic systems [16].

Advanced DLW processes have enabled the realization of diffractive lenses and metalenses using distinct wavefront-engineering strategies [16]. One such approach harnesses the Pancharatnam–Berry phase modulation, which employs in-plane rotation of the unit cells to achieve broadband, polarization-sensitive phase control while maintaining minimal thickness and high-resolution fabrication [17]. Similarly, geometrical tuning of the nanostructure dimensions, by adjusting height and diameter, allows the precise manipulation of the effective refractive index and phase delay, resulting in high-efficiency, achromatic focusing [18]. DLW also facilitates complex and highly resolved diffractive designs allowing multi-foci architectures. Involving a sparse-aperture configuration, each focal plane is produced by a spatially multiplexed array of square-nanohole metalenses. This design generates polarization-independent multiple focal planes with performances comparable to single-focus metalenses [19]. Beyond this, Balli et al. presented a rotationally tunable varifocal doublet metasurfaces, which exploits mutually rotated 3D-printed singlet elements to dynamically tune the focal lengths with quadratic radial phase profiles [20]. Furthermore, a single-layer aberration-compensated lens realized by combining multilevel supercritical patterns with an aberration-correction phase, forming concentric multilevel belts, enables sub-diffraction-limited focusing while compensating off-axis aberrations across an extended angular field [21].

Building on that, the versatility of DLW in fabricating subwavelength metastructures has enabled a broad spectrum of advanced photonic applications. For example, optical trapping benefits from metafibers produced through this technique, which feature ultrahigh numerical apertures, achieve diffraction-limited focal spots directly on fiber facets and enable flexible manipulation of microbeads and bacteria with a single optical fiber [22]. In telecommunication and endoscopic imaging, 3D-printed achromatic metalenses on single-mode fiber tips provide broadband, polarization-insensitive focusing across the entire near-infrared range, compensating chromatic aberrations and supporting high-resolution confocal imaging, wavelength-multiplexing communications, and femtosecond-laser-assisted procedures [23]. The precise control afforded by DLW also allows dual-wavelength and multidimensional holography, where propagation and geometric phases are combined to achieve accurate phase control at multiple visible wavelengths for applications in holographic displays, optical encryption, and anti-counterfeiting [24]. Furthermore, inverse-designed metalenses fabricated by DLW on fiber tips convert collimated input wavefronts into tightly focused spherical spots at submicron scales, enabling high-precision 3D microfabrication [25]. Minimally invasive endoscopic imaging is also advanced through ultrathin, aberration-corrected freeform micro-optics printed on single-mode fibers, producing functional optical coherence tomography probes with diameters below 0.5 mm while maintaining high-resolution structural imaging [26].

Collectively, these examples highlight how DLW-driven micro-optics facilitate multifunctional, compact, and high-performance photonic devices across biomedical, communication, and precision fabrication applications.
